# Methods for analysing the relationship between poverty, parental work intensity, child emotional symptoms and conduct problems over time

**DOI:** 10.1016/j.mex.2022.101940

**Published:** 2022-12-02

**Authors:** Patricio Troncoso, Morag Treanor

**Affiliations:** Heriot-Watt University, Edinburgh Campus, Edinburgh, Scotland EH14 4AS, United Kingdom

**Keywords:** Poverty, Parental work intensity, Children's mental health, Emotional wellbeing, Conduct problems, Growing Up in Scotland, Multivariate Multilevel Modelling, Growth Curve Modelling, Longitudinal Measurement Invariance Growth mixture modelling Multivariate Growth Curve Modelling

## Abstract

This article exposes the methods employed to analyse the complex associations between poverty and work intensity over time on the longitudinal trajectories of mental health wellbeing in a cohort of children. This study used data from nine waves of birth cohort 1 of the Growing Up in Scotland (GUS) study (2005/06–2017/18) to fit a bivariate multilevel non-linear growth curve model for the change in conduct problems and emotional symptoms of children over time with the trajectories of poverty and parental work intensity over time as the main covariates of interest. We explain in detail: (a) how we arrive at valid measures for our outcome of interest by testing for longitudinal measurement invariance and (b) the principled approach of growth mixture modelling undertaken to derive our main covariates of interest. Both procedures are the preamble for the main model of interest that addresses the substantive research question of how changes over time in poverty and parental employment are associated with changes over time in children's wellbeing.

• We expose the rationale behind and the procedures for implementing Longitudinal Measurement Invariance testing for the repeated measures of emotional and conduct problems.
• We expose the rationale behind and the procedures for implementing a growth mixture modelling approach to derive longitudinal measures of poverty and work intensity.
• We provide details of the bivariate growth curve model fitted to analyse the effect of the derived longitudinal measures of poverty and work intensity on the valid longitudinal measures of emotional and conduct problems.

## Graphical abstract

**Figure:**
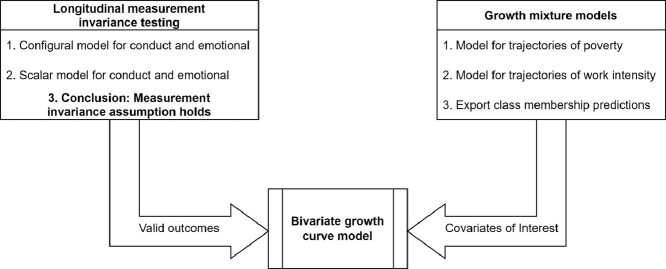

Specifications table

**Table:** 

Subject area:	Medicine and Dentistry
More specific subject area:	*Social epidemiology*
Name of your method:	Longitudinal Measurement Invariance Growth mixture modelling Multivariate Growth Curve Modelling
Name and reference of original method:	*Grimm, K., Ram, N., & Estabrook, R. (2017). Growth modeling. Structural equation and multilevel modeling approaches. The Guilford Press. Muthén B. (2004). Latent variable analysis. Growth mixture modeling and related techniques for longitudinal data. In: Kaplan D, editor. The Sage handbook of quantitative methodology for the social sciences. Thousand Oaks: Sage Publications. pp. 345–368. Goldstein, H. (2011). Multilevel statistical models (4th ed.). John Wiley and Sons, Ltd.*
Resource availability:	*Access to the data necessary to reproduce the results can be requested via the UK Data Service (*https://doi.org/10.5255/UKDA-SN-5760-12*). The methods described here are implemented using the following software: R packages “lavaan” and “R2MLwiN”, MLwiN and Latent Gold. Full details of the code are provided.*

## Method details

### Introduction and background

The methods presented here correspond to the procedures adopted by Treanor and Troncoso [11] to address the various complexities associated with analysing the longitudinal interrelationships between poverty, parental work intensity, child emotional symptoms and conduct problems.

This work utilised data from the first birth cohort of the “Growing up in Scotland” (GUS) longitudinal survey [10]. In this survey, 5,217 children, born in 2004/05 in Scotland, have been followed over time for 9 waves (at the time of writing) from 2005/06 to 2017/18. The variables selected correspond to: (a) children's emotional symptoms and conduct problems subscales of the “Strengths and Difficulties Questionnaire” (SDQ) [5]; (b) poverty as derived from official poverty thresholds based on income; (c) work intensity status as derived from marriage/partnership status and employment status; and (d) demographic variables.

Firstly, children's emotional symptoms and conduct problems are repeated measures, but the mere fact that the same instruments are applied at each point is not a guarantee that the observed data is indeed a realisation of the same construct over time. In these circumstances, it is necessary to deploy a method that allows the researchers to determine whether the obtained measurements maintain a certain structure over time. This is the rationale behind longitudinal measurement invariance testing, which is the focus of Section 2 of this article.

Second, socio-economic circumstances are time-varying, and even though they can be easily incorporated in a multilevel longitudinal model (as level-1 variables), we hypothesised that the overall trajectories of socio-economic status can provide further nuance than point-in-time states cannot. For instance, as Treanor and Troncoso [11] argue, a status of poor at any given point in time can be the manifestation of at least 2 different overall trajectories; i.e. falling into poverty or escaping it, but it can also reflect a short-lived spell of poverty that does not repeat itself. With repeated measures over time, we can estimate a model which can account for different patterns over time and cluster individuals around plausible trajectory groups. This is the rationale behind using the growth mixture models for longitudinal poverty and work intensity that we describe in Section 3.

Finally, we describe in Section 4 the model we fitted for the effect of the estimated latent classes of longitudinal poverty and work intensity (as described in Section 3) on the longitudinal trajectories of conduct problems and emotional symptoms. This model has two outcome variables and we have treated time flexibly with polynomials, hence this is a bivariate non-linear growth curve model.

### Longitudinal measurement invariance

We performed item-level analyses of SDQ items [5] to determine the longitudinal invariance of the conduct and emotional problems subscales, following Grimm et al. [6] approach. The procedure involved replicating the factor structure over the six time points (waves 4 to 9) for each of the selected SDQ subscales (conduct and emotional) and fitting two models to evaluate the fit of the measurement model with and without constraints. The specific items used are presented in Appendix 1.

The first measurement model was an unconstrained model, that is, with freely estimated factor loadings, thresholds, variances and covariances, but keeping the hypothesised factorial structure and relationships between items over time. This model is what we term “configural invariance” (m1) and we used it as the baseline comparison for the constrained model. The “scalar invariance” model (m2) constrained the factor loadings and thresholds across time points to equality. The results for these models are displayed in Tables 1 and 2. Some standardised factor loadings are greater than one; however, these are not problematic or unexpected for two main reasons: (a) the items contained in each factor are indeed known to be correlated, which is a prerequisite for Confirmatory Factor Analysis; and (b) the items are repeated measures over time and hence the factors were allowed to be correlated (oblique), therefore factor loadings are regression coefficients and not correlations. For a more detailed discussion on this issue, see: Deegan [2].

**Table 1:** Scalar invariance model for conduct problems over time.

Factor loadings				
Wave	Item	Coef.	SE	Std. Coef.
4	sdq5	1.000	0.000	0.945
	sdq7	0.815	0.045	0.770
	sdq12	0.987	0.064	0.933
	sdq18	0.791	0.042	0.747
	sdq22	0.768	0.061	0.726

5	sdq5	1.000	0.000	0.939
	sdq7	0.815	0.045	0.765
	sdq12	0.987	0.064	0.926
	sdq18	0.791	0.042	0.742
	sdq22	0.768	0.061	0.721

6	sdq5	1.000	0.000	0.955
	sdq7	0.815	0.045	0.778
	sdq12	0.987	0.064	0.942
	sdq18	0.791	0.042	0.755
	sdq22	0.768	0.061	0.734

7	sdq5	1.000	0.000	1.024
	sdq7	0.815	0.045	0.835
	sdq12	0.987	0.064	1.010
	sdq18	0.791	0.042	0.810
	sdq22	0.768	0.061	0.787

8	sdq5	1.000	0.000	0.993
	sdq7	0.815	0.045	0.809
	sdq12	0.987	0.064	0.979
	sdq18	0.791	0.042	0.785
	sdq22	0.768	0.061	0.763

9	sdq5	1.000	0.000	1.064
	sdq7	0.815	0.045	0.867
	sdq12	0.987	0.064	1.049
	sdq18	0.791	0.042	0.841
	sdq22	0.768	0.061	0.817


Thresholds (constrained to equality over time)				
Item	Threshold	Coef.	SE	Std. Coef.
sdq5	1	-0.129	0.021	-0.129
	2	1.493	0.032	1.493

sdq7	1	0.038	0.018	0.038
	2	2.197	0.032	2.197

sdq12	1	2.021	0.050	2.021
	2	3.364	0.079	3.364

sdq18	1	1.167	0.025	1.167
	2	2.772	0.045	2.772

sdq22	1	2.525	0.065	2.525
	2	3.229	0.086	3.229

*Note:* Unstandardised factor loadings are constrained to equality over time. All p-values are lower than 0.05.

**Table 2:** Scalar invariance model for emotional symptoms over time.

Factor loadings				
Wave	Item	Coef.	SE	Std. Coef.
4	sdq3	1.000	0.000	0.417
	sdq8	2.434	0.139	1.015
	sdq13	1.864	0.105	0.777
	sdq16	1.580	0.086	0.659
	sdq24	2.354	0.129	0.982

5	sdq3_5	1.000	0.000	0.442
	sdq8_5	2.434	0.139	1.075
	sdq13_5	1.864	0.105	0.823
	sdq16_5	1.580	0.086	0.698
	sdq24_5	2.354	0.129	1.040

6	sdq3_6	1.000	0.000	0.487
	sdq8_6	2.434	0.139	1.184
	sdq13_6	1.864	0.105	0.907
	sdq16_6	1.580	0.086	0.769
	sdq24_6	2.354	0.129	1.146

7	sdq3_7	1.000	0.000	0.561
	sdq8_7	2.434	0.139	1.365
	sdq13_7	1.864	0.105	1.045
	sdq16_7	1.580	0.086	0.886
	sdq24_7	2.354	0.129	1.320

8	sdq3_8	1.000	0.000	0.624
	sdq8_8	2.434	0.139	1.518
	sdq13_8	1.864	0.105	1.163
	sdq16_8	1.580	0.086	0.986
	sdq24_8	2.354	0.129	1.469

9	sdq3_9	1.000	0.000	0.719
	sdq8_9	2.434	0.139	1.749
	sdq13_9	1.864	0.105	1.339
	sdq16_9	1.580	0.086	1.135
	sdq24_9	2.354	0.129	1.692


Thresholds (constrained to equality over time)				
Item	Threshold	Coef.	SE	Std. Coef.
sdq3	1	0.903	0.019	0.903
	2	2.065	0.029	2.065

sdq8	1	1.274	0.036	1.274
	2	3.073	0.068	3.073

sdq13	1	1.687	0.036	1.687
	2	3.098	0.056	3.098

sdq16	1	0.378	0.019	0.378
	2	1.974	0.029	1.974

sdq24	1	1.027	0.030	1.027
	2	2.914	0.056	2.914

*Note:* Unstandardised factor loadings are constrained to equality over time. All p-values are lower than 0.001.

We used the R package “lavaan” [9] to run confirmatory factor analyses (CFA) for ordered categorical outcomes. This is done via the Mean and Variance Adjusted Weighted Least Squares (WLSMV) algorithm. The complete code to replicate these analyses is given in Appendix 2.

Goodness of fit was assessed by using the Comparative Fit Index (CFI), Tucker Lewis Index (TLI) and the Root Mean Square Error Approximation (RMSEA). Chi-squared values are also reported, but its oversensitivity over large samples should be noted. Scaled goodness of fit measures are also reported given the models were fitted via the WLSMV algorithm. The configural (unconstrained) measurement models yielded better fit than the scalar invariance (constrained) models for both outcomes. Nevertheless, scalar invariance models did yield excellent fit, with CFI and TLI values over 0.95 and RMSEA values comfortably below the conventional threshold, i.e. <0.06 [7]. A comparison of the model fit of these models for all outcomes can be seen in Table 3.

**Table 3:** Goodness of fit comparison of longitudinal measurement invariance models for conduct and emotional problems.

Outcome	Invariance model	CFI (scaled)	TLI (scaled)	RMSEA (scaled)	Chi-squared (scaled)	df
Conduct (a)	Configural (m1a)	0.994 (0.983)	0.991 (0.977)	0.021 (0.023)	848.4 (971.2)	315
	Scalar (m2a)	0.967 (0.944)	0.963 (0.937)	0.042 (0.038)	3159.3 (2563.4)	385

Emotional (b)	Configural (m1b)	0.996 (0.987)	0.995 (0.982)	0.018 (0.021)	701.01 (878.9)	315
	Scalar (m2b)	0.978 (0.954)	0.975 (0.948)	0.038 (0.036)	2607.3 (2373.5)	385

*Note:* CFI = Comparative Fit Index; TLI = Tucker Lewis Index; RMSEA = Root Mean Square Error of Approximation; df = degrees of freedom.

In conclusion, it is reasonable to assume scalar longitudinal measurement invariance since the models for both outcomes showed adequate fit. This allowed us to use the SDQ subscale composite scores, because measurement invariance avoids confounding change in scores over time with changes in reliability of the items [8].

### Growth mixture models

The variables related to work intensity and longitudinal poverty variables over time were used to fit two separate growth mixture models [16] to identify latent classifications of children. These models were fitted in Latent Gold version 6.0 [14] via maximum a posteriori (MAP) estimation, which is used to avoid boundary solutions, as described in Galindo Garrido and Vermunt [3], as well as Vermunt and Magidson [14]. The models are fitted with all available information across all the waves for which there is data on the observed variables that compose the two latent class models; this is 8 waves for longitudinal poverty and 9 for work intensity. The complete code to run these models is given in Appendix 2.

#### Longitudinal poverty

This growth mixture model uses the poverty binary indicator (coded as 1 = poor; 0 = non-poor), which is available for sweeps 1 to 8 of the GUS data. As such, the model is fitted as a binary logistic growth mixture model. The results are presented in Table 4.

**Table 4:** Parameters of the growth mixture model for longitudinal poverty.

Term	Class	Coef.	s.e.	p
Initial score	1	-4.253	0.305	0.000
Initial score	2	-0.031	1.086	0.980
Initial score	3	1.874	0.257	0.000
Initial score	4	-2.503	0.487	0.000

Growth rate	1	-0.479	0.131	0.000
Growth rate	2	-0.207	0.268	0.440
Growth rate	3	-0.010	0.029	0.730
Growth rate	4	0.260	0.080	0.001

Intercept	1	0.884	0.063	0.000
Intercept	2	-0.754	0.632	0.230
Intercept	3	0.144	0.122	0.240
Intercept	4	-0.274	0.503	0.590

Variance (Initial score)	Overall	3.984	1.487	
Covariance (Initial-Growth)	Overall	-0.432	0.182	
Variance (Growth rate)	Overall	0.047	0.024	

Model fit information	Value			

N	5217			
Number of parameters	14			
Log-likelihood	-11051.23			
BIC	22222.30			
AIC	22130.47			
Entropy	0.492			

**Fig. 1:**
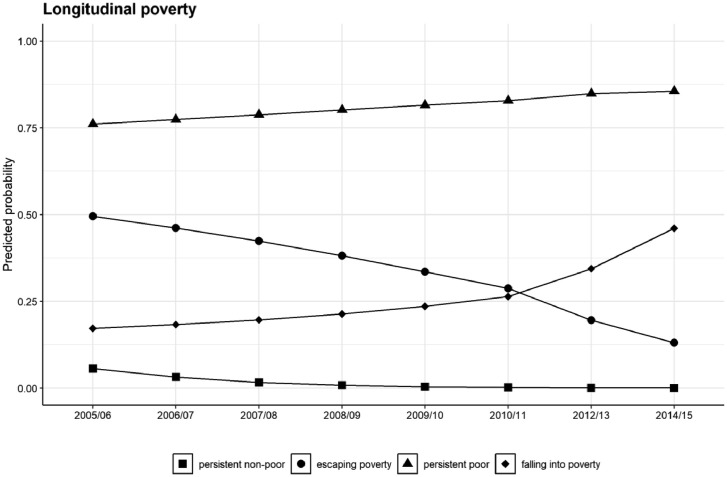
Trajectories of the predicted probabilities of being poor by latent class.

This model includes four latent classes, which we compared against competing models with two, three and five latent classes. Estimated coefficients are in the logit scale and given that this is a longitudinal model, observing the overall trajectories are what matters most to understand the different patterns that the underlying groups undergo. Fig. 1 presents the predicted trajectories of each of these four classes in terms of the probability of the individuals of being poor.

Class 1 starts off with a low probability of being in poverty and this decreases slightly with time, which is why we denominated this class as “Persistent non-poor”. Class 2, on the other hand, starts off with an intermediate probability of being under the poverty line (about 0.5), but rapidly decreases well below 0.25; this is a class characterised by a process of “Escaping poverty”. With regard to class 3, we observe a high probability of being in poverty since the beginning (just over 0.75) and even slightly increasing over time; this is the class we termed as the “Persistent poor”. Finally, class 4 starts off with a probability just under 0.25 and increases slowly over time until reaching the year 2010/11, where a more pronounced increase is observed; we have denominated this class as “Falling into poverty”.

#### Work intensity

This growth mixture model uses a custom continuous index of work intensity, which ranges from 0 to 1 and is constructed by combining the employment information of the main respondent (usually the mother) and their partners (if present). For a couple family, the range is: 1 = both partners in full-time work; 0.75 = one full-time and one part-time partner; 0.5 = one full-time or two part-time partners; and 0.25 = one part-time partner, one partner not in paid work. For a lone parent the range is: 1 = lone parent working full-time; 0.5 = lone parent working part-time; and 0 = lone parent not working. This means that a full-time working lone parent has the same weighting as a full-time working couple. This information is available for sweeps 1 to 9 of the GUS data. The model is fitted as a growth mixture model for continuous responses. The results are presented in Table 5.

**Table 5:** Parameters of the growth mixture model for work intensity.

Term	Class	Coef.	s.e.	p
Initial score	1	0.864	0.032	0.000
Initial score	2	5.097	0.549	0.000
Initial score	3	-5.514	0.489	0.000
Initial score	4	-0.280	0.355	0.430
Initial score	5	-13.642	1.016	0.000

Growth rate	1	0.055	0.004	0.000
Growth rate	2	1.476	0.154	0.000
Growth rate	3	0.508	0.058	0.000
Growth rate	4	-0.698	0.105	0.000
Growth rate	5	0.785	0.119	0.000

Intercept	1	2.212	0.039	0.000
Intercept	2	-0.193	0.060	0.001
Intercept	3	-0.425	0.121	0.000
Intercept	4	-1.339	0.115	0.000
Intercept	5	-0.254	0.091	0.005

Variance (Initial score)	Overall	0.690	0.071	
Covariance (Initial-Growth)	Overall	0.011	0.004	
Variance (Growth rate)	Overall	0.000	0.000	

Model fit information	Value			

N	5217			
Number of parameters	17			
Log-likelihood	-48637.3			
BIC	97420.08			
AIC	97308.56			
Entropy	0.705			

**Fig. 2:**
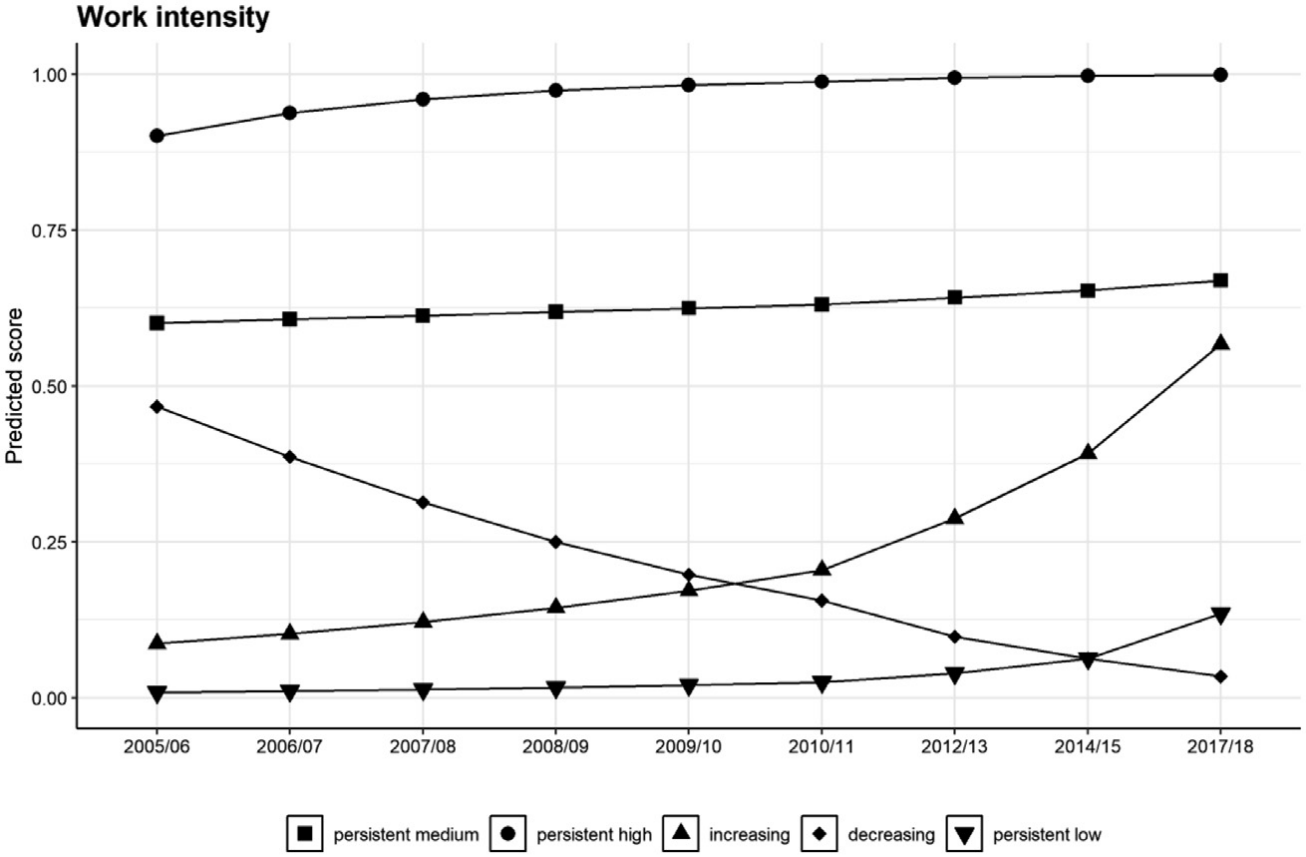
Trajectories of the predicted scores of work intensity by latent class.

This model includes five latent classes, which we compared against competing models with two, three and four latent classes. As was the case of longitudinal poverty in Section 3.1, observing the predicted trajectories of each of these five classes in terms of their predicted work intensity scores, as presented in Fig. 2, is a more convenient way of characterizing the longitudinal patterns that the latent groups (classes) undergo.

Class 1 is characterised by a relatively stable trajectory in the middle range of work intensity (0.5), which is why we denominated this class as “Persistent medium”. Class 2 starts off at relatively high value of work intensity and rapidly approaches the maximum intensity (1); this is the class denominated as “Persistent high”. On another front, class 3 is at the low values of work intensity at the beginning of the study period, but sharply increases from around 2009/10 until nearly catching up with the persistent medium class, which is why we denominated this class as “Increasing”. On the contrary, class 4 undergoes the opposite process, starting off at the medium values of intensity and plunging even below the persistent low intensity class; this is the class labelled as “Decreasing”. Finally, class 5 remains for most of the period of study at the very low values of work intensity, only increasing slightly by the end, which is why we termed this class as “Persistent low” work intensity.

### Bivariate multilevel non-linear growth curve model

We chose to model the effect of the longitudinal trajectories of poverty and work intensity on the longitudinal trajectories of conduct problems and emotional symptoms using a multivariate multilevel modelling framework. As pointed out by Troncoso [12] and Troncoso and Humphrey [13], this framework enabled us to analyse trajectories that: have a pattern over time; have varying growth rates across children; vary according to individual characteristics and; are specific to either conduct problems or emotional symptoms. The equation below follows the general multilevel notation as described in Goldstein [4].

$$y_{ij}=\beta_{01}z_{1ij}+\beta_{02}z_{2ij}+\beta_{11j}z_{1ij}age_{ij}+\beta_{12j}z_{2ij}age_{ij}+\beta_{21}z_{1ij}age_{ij}^{2}+\beta_{22}z_{2ij}age_{ij}^{2}\;\beta_{01ij}=\beta_{01}+u_{01j}+e_{01ij}$$

$$\beta_{02ij}=\beta_{02}+u_{02j}+e_{02ij}$$

$$\beta_{11j}=\beta_{11}+u_{11j}$$

$$\beta_{12j}=\beta_{12}+u_{12j}$$

where:

$$z_{1ijk}=\left\{\begin{array}{c} 1\;if\;conduct\\ 0\;otherwise \end{array}\right\},\;z_{2ijk}=\left\{\begin{array}{c} 1\;if\;emotional\\ 0\;otherwise \end{array}\right\}$$

$$\left[\begin{array}{c} \begin{array}{c} u_{01j}\\ u_{02j}\\ u_{11j} \end{array}\\ u_{12j} \end{array}\right]∼MVN\left(0,\Omega_{u}\right):\Omega_{u}=\left[\begin{array}{cc} \begin{array}{cc} \sigma_{u01}^{2} & \sigma_{u01,02}\\ \sigma_{u01,02} & \sigma_{u02}^{2} \end{array} & \begin{array}{cc} \sigma_{u01,11} & \sigma_{u01,12}\\ \sigma_{u02,11} & \sigma_{u02,12} \end{array}\\ \begin{array}{cc} \sigma_{u01,11} & \sigma_{u02,11}\\ \sigma_{u01,12} & \sigma_{u02,12} \end{array} & \begin{array}{cc} \sigma_{u11}^{2} & \sigma_{u11,12}\\ \sigma_{u11,12} & \sigma_{u12}^{2} \end{array} \end{array}\right]$$

$$\left[\begin{array}{c} e_{01ij}\\ e_{02ij} \end{array}\right]∼MVN\left(0,\Omega_{e}\right):\Omega_{e}=\left[\begin{array}{cc} \sigma_{e01}^{2} & \sigma_{e01,02}\\ \sigma_{e01,02} & \sigma_{e02}^{2} \end{array}\right]$$

$y_{ij}$ is a twofold set of outcome variables defined by the dummy variables $z_{1ij}$ (conduct problems) and $z_{2ij}$ (emotional symptoms). The data set has a long format with two observations per case, which adds an artificial level to fit two equations simultaneously. The subscripts “i” and “j” denote the levels of occasions (time) and children, respectively. $\beta_{01}$ and $\beta_{02}$ correspond to the intercepts of each measure, which are allowed to vary randomly at the levels of children. $\beta_{11j}$ and $\beta_{12j}$ are the growth rates for each of the outcome measures, which are allowed to vary randomly across children. The growth rates have the associated errors denoted by $u_{11j}$ and $u_{12j}$. Time (age) is treated flexibly via the addition of fixed squared terms, whose effects are denoted by the set of coefficients $\beta_{21}$ and $\beta_{22}$. The fixed part of the model also contains a further set of covariates which specified in the full model but are omitted here for simplicity. The random part of the model is split into two variance-covariance matrices $\Omega_{u}$ and $\Omega_{e}$, which correspond to the levels of children and occasions, respectively. Each diagonal element of matrix $\Omega_{e}$ corresponds to the variances of the intercepts of the two outcomes at the occasion level, while the off-diagonal elements are the covariances between them. In matrix $\Omega_{u}$, the first two diagonal elements are the intercepts for the outcomes and the last two are the variances of the slopes of the linear terms for time (growth rates), while its off-diagonal elements correspond to the covariances between the intercepts and the slopes. The code to run the model described above is given in Appendix 4.

Treanor and Troncoso [11] used this model and incorporated the longitudinal poverty and work intensity classes as the main covariates of interest, along with other control variables. The results of the full model are reproduced here in Table 6.

**Table 6:** Bivariate multilevel growth curve model for conduct problems and emotional symptoms, controlling for work intensity and longitudinal poverty.

Main effects	Conduct				Emotional			
	Mean	SD	CI low	CI high	Mean	SD	CI low	CI high
Intercept	0.385	0.082	0.223	0.544	-0.179	0.073	-0.321	-0.035
Age	-0.226	0.020	-0.265	-0.186	0.003	0.021	-0.038	0.044
Age squared	0.052	0.007	0.039	0.066	0.011	0.007	-0.003	0.025
Age cubed	-0.005	0.001	-0.006	-0.003	-0.001	0.001	-0.002	0.001
Persistent medium intensity	0.005	0.052	-0.096	0.108	0.065	0.044	-0.023	0.152
Persistent low intensity	0.169	0.091	-0.007	0.347	0.026	0.078	-0.127	0.179
Increasing intensity	0.194	0.091	0.018	0.375	0.075	0.078	-0.077	0.228
Decreasing intensity	-0.064	0.126	-0.311	0.189	0.212	0.107	0.003	0.422
Falling into poverty	0.101	0.043	0.017	0.184	0.026	0.037	-0.046	0.100
Escaping poverty	0.234	0.070	0.095	0.370	0.096	0.060	-0.020	0.213
Persistently poor	0.214	0.047	0.122	0.306	0.078	0.041	-0.002	0.157
Material deprivation	0.105	0.014	0.077	0.133	0.120	0.013	0.095	0.146
Vocational qualification	0.072	0.029	0.015	0.129	0.025	0.026	-0.026	0.076
Higher grade	-0.045	0.053	-0.148	0.058	-0.067	0.048	-0.161	0.026
Standard grade	0.154	0.042	0.070	0.237	0.067	0.039	-0.009	0.143
Other qualifications	0.536	0.272	0.004	1.067	0.325	0.239	-0.134	0.798
No qualifications	0.253	0.060	0.135	0.371	0.132	0.056	0.022	0.242
Mother's age 20–29	-0.137	0.064	-0.260	-0.013	-0.026	0.057	-0.138	0.086
Mother's age 30–39	-0.158	0.064	-0.284	-0.032	-0.099	0.058	-0.213	0.015
Mother's age 40+	-0.254	0.090	-0.429	-0.079	-0.085	0.080	-0.242	0.072
Non-White ethnicity	-0.019	0.068	-0.154	0.114	0.113	0.061	-0.007	0.233
Female child	-0.191	0.024	-0.238	-0.143	-0.006	0.022	-0.049	0.037

Interactions	Mean	SD	CI low	CI high	Mean	SD	CI low	CI high

Age*Persistent medium intensity	0.003	0.010	-0.016	0.023	-0.001	0.011	-0.023	0.021
Age*Persistent low intensity	0.030	0.019	-0.008	0.068	0.068	0.021	0.026	0.110
Age*Increasing intensity	-0.012	0.018	-0.048	0.024	0.036	0.021	-0.004	0.076
Age*Decreasing intensity	0.079	0.025	0.029	0.128	0.019	0.028	-0.035	0.073
Age*Falling into poverty	0.006	0.008	-0.010	0.023	0.021	0.009	0.003	0.039
Age*Escaping poverty	-0.006	0.014	-0.033	0.021	-0.002	0.015	-0.032	0.028
Age*Persistently poor	-0.003	0.009	-0.021	0.014	0.000	0.010	-0.020	0.019

*Notes:* Reference categories = Persistent high intensity, persistently non-poor, University degree, Mother's age under 20, White ethnicity, male. Parameters were obtained via MCMC using 2 chains of length 15,000. All fixed-effects parameters have an effective sample size (ESS) of at least 2,000. Deviance information criterion = 67,692.848. The model uses diffuse prior distributions as described in Browne [1].

It is beyond the scope of this article to discuss the results in detail, hence interested readers are advised to consult Treanor and Troncoso [11] for interpretation of these model coefficients and contrast with the relevant literature. A summary of these findings is also provided in the next section.

## Conclusion

By applying the methods described here, Treanor and Troncoso [11] were able to conclude the following: (1) conduct problems tend to decrease over time as children age, but at varying rates depending on children's characteristics; (2) emotional problems tend to increase over time and become more severe as children age; (3) children in families who are persistently poor, escaping poverty, or falling into poverty have increased rates of conduct problems; (4) children's conduct problems tend to increase over time and become more severe as they age; (5) children whose parents have decreasing work intensity have significantly higher rates of emotional symptoms than those with persistently high intensity; and (6) children whose parents have persistently high and medium work intensity have among the lowest scores for both conduct and emotional problems, indicating that income and employment stability is beneficial to children's mental health.

Overall, these analyses allow emphasising that children do not exist in isolation but rather as members of families, whose economic circumstances affect them both directly and indirectly. It is therefore necessary to maintain young people at the centre of policies to ensure their protection against the multiple, i.e., social and health, harms of economic crises.

## CRediT authorship contribution statement

**Patricio Troncoso:** Methodology, Formal analysis, Data curation, Visualization, Writing – original draft, Writing – review & editing. **Morag Treanor:** Conceptualization, Methodology, Supervision, Writing – original draft, Writing – review & editing, Project administration.

## Declaration of Competing Interest

The authors declare that they have no known competing financial interests or personal relationships that could have appeared to influence the work reported in this paper.

## Figures and Tables

**Table:** 

options
algorithm emiterations=250 nriterations=50;
startvalues seed=0 sets=100 iterations=250;
bayes categorical=10 variances=10
latent=10 poisson=10; // for MAP estimation
quadrature nodes=10;
missing includeall; // to keep all available information
output
parameters=effect betaopts=wl
standarderrors=robust
profile probmeans=posterior
estimatedvalues=regression
iterationdetails classification;
outfile "[path/filename].sav" // filename for class membership predictions
classification=posterior;
variables
caseid id;
dependent poor.1 binomial, poor.2 binomial,
poor.3 binomial, poor.4 binomial,
poor.5 binomial, poor.6 binomial,
poor.7 binomial, poor.8 binomial;
latent
Class nominal 4,
i continuous,
s continuous;
equations
Class <- 1;
i;
s;
i <-> s;
i <- 1|Class;
s <- 1|Class;
poor.1 <- (1) i + (0) s;
poor.2 <- (1) i + (1) s;
poor.3 <- (1) i + (2) s;
poor.4 <- (1) i + (3) s;
poor.5 <- (1) i + (4) s;
poor.6 <- (1) i + (5) s;
poor.7 <- (1) i + (7) s;
poor.8 <- (1) i + (9) s;

**Table:** 

options
algorithm emiterations=250 nriterations=50;
startvalues seed=0 sets=100 iterations=250;
bayes categorical=10 variances=10
latent=10 poisson=10; // for MAP estimation
quadrature nodes=10;
missing includeall; // to keep all available information
output
parameters=effect betaopts=wl
standarderrors=robust
profile probmeans=posterior
estimatedvalues=regression
iterationdetails classification;
outfile "[path/filename].sav" // filename for class membership predictions
classification=posterior;
variables
caseid id;
dependent wint.1, wint.2, wint.3,
wint.4, wint.5, wint.6,
wint.7, wint.8, wint.9;
latent
Class nominal 5,
i continuous,
s continuous;
equations
Class <- 1;
i;
s;
i <-> s;
i <- 1|Class;
s <- 1|Class;
wint.1 <- (1) i + (0) s;
wint.2 <- (1) i + (1) s;
wint.3 <- (1) i + (2) s;
wint.4 <- (1) i + (3) s;
wint.5 <- (1) i + (4) s;
wint.6 <- (1) i + (5) s;
wint.7 <- (1) i + (7) s;
wint.8 <- (1) i + (9) s;
wint.9 <- (1) I + (12) s;

**Table:** 

model <- runMLwiN(c(conduct, emotional) ∼ 1 + age + I(age^2) + # fixed part
(1 + age | id) + (1 | waveid), # random part
D=c("Multivariate Normal"),
data = data,
estoptions = list(EstM = 1)) # this is for MCMC estimation

**Table:** 

full_model <- runMLwiN(c(conduct, emotional) ∼ 1 + age + I(age^2) +
factor(poverty) + factor(work) + # main covariates
factor(education) + factor(m_age) + # controls
factor(ethnicity) + factor(sex) + # controls
age*factor(poverty) + age*factor(work) + # interactions
(1 + age | id) + (1 | waveid), # random part
D=c("Multivariate Normal"),
data = data,
estoptions = list(EstM = 1)) # this is for MCMC estimation

## Data Availability

The authors do not have permission to share data.

## Appendix 1 SDQ Items used in this study

We have used the SDQ subscales of “conduct problems” and “emotional symptoms”, for the reasons explained in Treanor and Troncoso [11]. The items below are reproduced from the GUS documentation [10]. Respondents (main carers) are given the following instructions: “Please give your answers on the basis of ^Childname‘s behaviour over the last six months.” All items have the same alternatives: 1 = “Not true”; 2 = “Somewhat true”; 3 = “Certainly true”; and 4 = “Can't say”. The original SDQ questionnaires with all the subscales are retrievable from: https://www.sdqinfo.org/py/sdqinfo/b0.py.

**Items of the Conduct problems subscale:**

SDQ5: ^Childname often has temper tantrums or hot tempers
SDQ7: ^Childname is generally obedient, usually does what adults request
SDQ12: ^Childname often fights with other children or bullies them
SDQ18: ^Childname often lies or cheats
SDQ22: ^Childname steals from home, school or elsewhere

**Items of the Emotional symptoms subscale:**

SDQ3: ^Childname often complains of headaches, stomach-aches or sickness
SDQ8: ^Childname has many worries, often seems worried
SDQ13: ^Childname is often unhappy, down-hearted or tearful
SDQ16: ^Childname is nervous or clingy in new situations, easily loses confidence
SDQ24: ^Childname has many fears, is easily scared

## Appendix 2 Longitudinal Measurement Invariance Models

The code below is for the R package “lavaan”. Comments are preceded by “#”, code chunks are separated by “#—”. Indentation is not required. Model specification must be within quotations (“”).

# ––––––––––––––––––

# Configural invariance conduct (m1a)

# ––––––––––––––––––

conf_cond <- "

# Factor models for conduct at 6 waves.

FX4 =∼ sdq5_4 + sdq7_4 + sdq12_4 + sdq18_4 + sdq22_4

FX5 =∼ sdq5_5 + sdq7_5 + sdq12_5 + sdq18_5 + sdq22_5

FX6 =∼ sdq5_6 + sdq7_6 + sdq12_6 + sdq18_6 + sdq22_6

FX7 =∼ sdq5_7 + sdq7_7 + sdq12_7 + sdq18_7 + sdq22_7

FX8 =∼ sdq5_8 + sdq7_8 + sdq12_8 + sdq18_8 + sdq22_8

FX9 =∼ sdq5_9 + sdq7_9 + sdq12_9 + sdq18_9 + sdq22_9

# thresholds conduct

sdq5_4 | t1 + t2

sdq5_5 | t1 + t2

sdq5_6 | t1 + t2

sdq5_7 | t1 + t2

sdq5_8 | t1 + t2

sdq5_9 | t1 + t2

sdq7_4 | t1 + t2

sdq7_5 | t1 + t2

sdq7_6 | t1 + t2

sdq7_7 | t1 + t2

sdq7_8 | t1 + t2

sdq7_9 | t1 + t2

sdq12_4 | t1 + t2

sdq12_5 | t1 + t2

sdq12_6 | t1 + t2

sdq12_7 | t1 + t2

sdq12_8 | t1 + t2

sdq12_9 | t1 + t2

sdq18_4 | t1 + t2

sdq18_5 | t1 + t2

sdq18_6 | t1 + t2

sdq18_7 | t1 + t2

sdq18_8 | t1 + t2

sdq18_9 | t1 + t2

sdq22_4 | t1 + t2

sdq22_5 | t1 + t2

sdq22_6 | t1 + t2

sdq22_7 | t1 + t2

sdq22_8 | t1 + t2

sdq22_9 | t1 + t2

# Covariances (observed conduct)

# item 5 all time points

 sdq5_4 ∼∼ sdq5_5

 sdq5_4 ∼∼ sdq5_6

 sdq5_4 ∼∼ sdq5_7

 sdq5_4 ∼∼ sdq5_8

 sdq5_4 ∼∼ sdq5_9

 sdq5_5 ∼∼ sdq5_6

 sdq5_5 ∼∼ sdq5_7

 sdq5_5 ∼∼ sdq5_8

 sdq5_5 ∼∼ sdq5_9

 sdq5_6 ∼∼ sdq5_7

 sdq5_6 ∼∼ sdq5_8

 sdq5_6 ∼∼ sdq5_9

 sdq5_7 ∼∼ sdq5_8

 sdq5_7 ∼∼ sdq5_9

 sdq5_8 ∼∼ sdq5_9

# item 7 all time points

 sdq7_4 ∼∼ sdq7_5

 sdq7_4 ∼∼ sdq7_6

 sdq7_4 ∼∼ sdq7_7

 sdq7_4 ∼∼ sdq7_8

 sdq7_4 ∼∼ sdq7_9

 sdq7_5 ∼∼ sdq7_6

 sdq7_5 ∼∼ sdq7_7

 sdq7_5 ∼∼ sdq7_8

 sdq7_5 ∼∼ sdq7_9

 sdq7_6 ∼∼ sdq7_7

 sdq7_6 ∼∼ sdq7_8

 sdq7_6 ∼∼ sdq7_9

 sdq7_7 ∼∼ sdq7_8

 sdq7_7 ∼∼ sdq7_9

 sdq7_8 ∼∼ sdq7_9

# item 12 all time points

 sdq12_4 ∼∼ sdq12_5

 sdq12_4 ∼∼ sdq12_6

 sdq12_4 ∼∼ sdq12_7

 sdq12_4 ∼∼ sdq12_8

 sdq12_4 ∼∼ sdq12_9

 sdq12_5 ∼∼ sdq12_6

 sdq12_5 ∼∼ sdq12_7

 sdq12_5 ∼∼ sdq12_8

 sdq12_5 ∼∼ sdq12_9

 sdq12_6 ∼∼ sdq12_7

 sdq12_6 ∼∼ sdq12_8

 sdq12_6 ∼∼ sdq12_9

 sdq12_7 ∼∼ sdq12_8

 sdq12_7 ∼∼ sdq12_9

 sdq12_8 ∼∼ sdq12_9

# item 18 all time points

 sdq18_4 ∼∼ sdq18_5

 sdq18_4 ∼∼ sdq18_6

 sdq18_4 ∼∼ sdq18_7

 sdq18_4 ∼∼ sdq18_8

 sdq18_4 ∼∼ sdq18_9

 sdq18_5 ∼∼ sdq18_6

 sdq18_5 ∼∼ sdq18_7

 sdq18_5 ∼∼ sdq18_8

 sdq18_5 ∼∼ sdq18_9

 sdq18_6 ∼∼ sdq18_7

 sdq18_6 ∼∼ sdq18_8

 sdq18_6 ∼∼ sdq18_9

 sdq18_7 ∼∼ sdq18_8

 sdq18_7 ∼∼ sdq18_9

 sdq18_8 ∼∼ sdq18_9

# item 22 all time points

 sdq22_4 ∼∼ sdq22_5

 sdq22_4 ∼∼ sdq22_6

 sdq22_4 ∼∼ sdq22_7

 sdq22_4 ∼∼ sdq22_8

 sdq22_4 ∼∼ sdq22_9

 sdq22_5 ∼∼ sdq22_6

 sdq22_5 ∼∼ sdq22_7

 sdq22_5 ∼∼ sdq22_8

 sdq22_5 ∼∼ sdq22_9

 sdq22_6 ∼∼ sdq22_7

 sdq22_6 ∼∼ sdq22_8

 sdq22_6 ∼∼ sdq22_9

 sdq22_7 ∼∼ sdq22_8

 sdq22_7 ∼∼ sdq22_9

 sdq22_8 ∼∼ sdq22_9

"

# Fit the model

conf_cond_fit <- cfa(conf_cond, data = datacond,

      estimator = "WLSMV", mimic = "mplus",

      parameterization = "theta", missing = "pairwise",

      ordered = datacond)

#––––––––––––––––––

# Scalar invariance conduct (m2a)

#––––––––––––––––––

scalar_cond <- "

# Constraints are needed for longitudinal measurement invariance

# Factor models for conduct at 6 waves (constrained).

FX4 =∼ a*sdq5_4 + b*sdq7_4 + c*sdq12_4 + d*sdq18_4 + e*sdq22_4

FX5 =∼ a*sdq5_5 + b*sdq7_5 + c*sdq12_5 + d*sdq18_5 + e*sdq22_5

FX6 =∼ a*sdq5_6 + b*sdq7_6 + c*sdq12_6 + d*sdq18_6 + e*sdq22_6

FX7 =∼ a*sdq5_7 + b*sdq7_7 + c*sdq12_7 + d*sdq18_7 + e*sdq22_7

FX8 =∼ a*sdq5_8 + b*sdq7_8 + c*sdq12_8 + d*sdq18_8 + e*sdq22_8

FX9 =∼ a*sdq5_9 + b*sdq7_9 + c*sdq12_9 + d*sdq18_9 + e*sdq22_9

# threshold constraints conduct

sdq5_4 | a1*t1 + a2*t2

sdq5_5 | a1*t1 + a2*t2

sdq5_6 | a1*t1 + a2*t2

sdq5_7 | a1*t1 + a2*t2

sdq5_8 | a1*t1 + a2*t2

sdq5_9 | a1*t1 + a2*t2

sdq7_4 | b1*t1 + b2*t2

sdq7_5 | b1*t1 + b2*t2

sdq7_6 | b1*t1 + b2*t2

sdq7_7 | b1*t1 + b2*t2

sdq7_8 | b1*t1 + b2*t2

sdq7_9 | b1*t1 + b2*t2

sdq12_4 | c1*t1 + c2*t2

sdq12_5 | c1*t1 + c2*t2

sdq12_6 | c1*t1 + c2*t2

sdq12_7 | c1*t1 + c2*t2

sdq12_8 | c1*t1 + c2*t2

sdq12_9 | c1*t1 + c2*t2

sdq18_4 | d1*t1 + d2*t2

sdq18_5 | d1*t1 + d2*t2

sdq18_6 | d1*t1 + d2*t2

sdq18_7 | d1*t1 + d2*t2

sdq18_8 | d1*t1 + d2*t2

sdq18_9 | d1*t1 + d2*t2

sdq22_4 | e1*t1 + e2*t2

sdq22_5 | e1*t1 + e2*t2

sdq22_6 | e1*t1 + e2*t2

sdq22_7 | e1*t1 + e2*t2

sdq22_8 | e1*t1 + e2*t2

sdq22_9 | e1*t1 + e2*t2

# Covariances (observed conduct)

# item 5 all time points

 sdq5_4 ∼∼ sdq5_5

 sdq5_4 ∼∼ sdq5_6

 sdq5_4 ∼∼ sdq5_7

 sdq5_4 ∼∼ sdq5_8

 sdq5_4 ∼∼ sdq5_9

 sdq5_5 ∼∼ sdq5_6

 sdq5_5 ∼∼ sdq5_7

 sdq5_5 ∼∼ sdq5_8

 sdq5_5 ∼∼ sdq5_9

 sdq5_6 ∼∼ sdq5_7

 sdq5_6 ∼∼ sdq5_8

 sdq5_6 ∼∼ sdq5_9

 sdq5_7 ∼∼ sdq5_8

 sdq5_7 ∼∼ sdq5_9

 sdq5_8 ∼∼ sdq5_9

# item 7 all time points

 sdq7_4 ∼∼ sdq7_5

 sdq7_4 ∼∼ sdq7_6

 sdq7_4 ∼∼ sdq7_7

 sdq7_4 ∼∼ sdq7_8

 sdq7_4 ∼∼ sdq7_9

 sdq7_5 ∼∼ sdq7_6

 sdq7_5 ∼∼ sdq7_7

 sdq7_5 ∼∼ sdq7_8

 sdq7_5 ∼∼ sdq7_9

 sdq7_6 ∼∼ sdq7_7

 sdq7_6 ∼∼ sdq7_8

 sdq7_6 ∼∼ sdq7_9

 sdq7_7 ∼∼ sdq7_8

 sdq7_7 ∼∼ sdq7_9

 sdq7_8 ∼∼ sdq7_9

# item 12 all time points

 sdq12_4 ∼∼ sdq12_5

 sdq12_4 ∼∼ sdq12_6

 sdq12_4 ∼∼ sdq12_7

 sdq12_4 ∼∼ sdq12_8

 sdq12_4 ∼∼ sdq12_9

 sdq12_5 ∼∼ sdq12_6

 sdq12_5 ∼∼ sdq12_7

 sdq12_5 ∼∼ sdq12_8

 sdq12_5 ∼∼ sdq12_9

 sdq12_6 ∼∼ sdq12_7

 sdq12_6 ∼∼ sdq12_8

 sdq12_6 ∼∼ sdq12_9

 sdq12_7 ∼∼ sdq12_8

 sdq12_7 ∼∼ sdq12_9

 sdq12_8 ∼∼ sdq12_9

# item 18 all time points

 sdq18_4 ∼∼ sdq18_5

 sdq18_4 ∼∼ sdq18_6

 sdq18_4 ∼∼ sdq18_7

 sdq18_4 ∼∼ sdq18_8

 sdq18_4 ∼∼ sdq18_9

 sdq18_5 ∼∼ sdq18_6

 sdq18_5 ∼∼ sdq18_7

 sdq18_5 ∼∼ sdq18_8

 sdq18_5 ∼∼ sdq18_9

 sdq18_6 ∼∼ sdq18_7

 sdq18_6 ∼∼ sdq18_8

 sdq18_6 ∼∼ sdq18_9

 sdq18_7 ∼∼ sdq18_8

 sdq18_7 ∼∼ sdq18_9

 sdq18_8 ∼∼ sdq18_9

# item 22 all time points

 sdq22_4 ∼∼ sdq22_5

 sdq22_4 ∼∼ sdq22_6

 sdq22_4 ∼∼ sdq22_7

 sdq22_4 ∼∼ sdq22_8

 sdq22_4 ∼∼ sdq22_9

 sdq22_5 ∼∼ sdq22_6

 sdq22_5 ∼∼ sdq22_7

 sdq22_5 ∼∼ sdq22_8

 sdq22_5 ∼∼ sdq22_9

 sdq22_6 ∼∼ sdq22_7

 sdq22_6 ∼∼ sdq22_8

 sdq22_6 ∼∼ sdq22_9

 sdq22_7 ∼∼ sdq22_8

 sdq22_7 ∼∼ sdq22_9

 sdq22_8 ∼∼ sdq22_9

"

# Fit the model

scalar_cond_fit <- cfa(scalar_cond, data = datacond,

      estimator = "WLSMV", mimic = "mplus",

      parameterization = "theta",

      missing = "pairwise", ordered = datacond)

# ––––––––––––––––––

# Configural invariance emotional (m1b)

# ––––––––––––––––––

conf_emo <- "

# Factor models for emotional at 6 waves.

FY4 =∼ sdq3_4 + sdq8_4 + sdq13_4 + sdq16_4 + sdq24_4

FY5 =∼ sdq3_5 + sdq8_5 + sdq13_5 + sdq16_5 + sdq24_5

FY6 =∼ sdq3_6 + sdq8_6 + sdq13_6 + sdq16_6 + sdq24_6

FY7 =∼ sdq3_7 + sdq8_7 + sdq13_7 + sdq16_7 + sdq24_7

FY8 =∼ sdq3_8 + sdq8_8 + sdq13_8 + sdq16_8 + sdq24_8

FY9 =∼ sdq3_9 + sdq8_9 + sdq13_9 + sdq16_9 + sdq24_9

# thresholds emotional

sdq3_4 | t1 + t2

sdq3_5 | t1 + t2

sdq3_6 | t1 + t2

sdq3_7 | t1 + t2

sdq3_8 | t1 + t2

sdq3_9 | t1 + t2

sdq8_4 | t1 + t2

sdq8_5 | t1 + t2

sdq8_6 | t1 + t2

sdq8_7 | t1 + t2

sdq8_8 | t1 + t2

sdq8_9 | t1 + t2

sdq13_4 | t1 + t2

sdq13_5 | t1 + t2

sdq13_6 | t1 + t2

sdq13_7 | t1 + t2

sdq13_8 | t1 + t2

sdq13_9 | t1 + t2

sdq16_4 | t1 + t2

sdq16_5 | t1 + t2

sdq16_6 | t1 + t2

sdq16_7 | t1 + t2

sdq16_8 | t1 + t2

sdq16_9 | t1 + t2

sdq24_4 | t1 + t2

sdq24_5 | t1 + t2

sdq24_6 | t1 + t2

sdq24_7 | t1 + t2

sdq24_8 | t1 + t2

sdq24_9 | t1 + t2

# Covariances (observed emotional)

# item 3 all time points

 sdq3_4 ∼∼ sdq3_5

 sdq3_4 ∼∼ sdq3_6

 sdq3_4 ∼∼ sdq3_7

 sdq3_4 ∼∼ sdq3_8

 sdq3_4 ∼∼ sdq3_9

 sdq3_5 ∼∼ sdq3_6

 sdq3_5 ∼∼ sdq3_7

 sdq3_5 ∼∼ sdq3_8

 sdq3_5 ∼∼ sdq3_9

 sdq3_6 ∼∼ sdq3_7

 sdq3_6 ∼∼ sdq3_8

 sdq3_6 ∼∼ sdq3_9

 sdq3_7 ∼∼ sdq3_8

 sdq3_7 ∼∼ sdq3_9

 sdq3_8 ∼∼ sdq3_9

# item 8 all time points

 sdq8_4 ∼∼ sdq8_5

 sdq8_4 ∼∼ sdq8_6

 sdq8_4 ∼∼ sdq8_7

 sdq8_4 ∼∼ sdq8_8

 sdq8_4 ∼∼ sdq8_9

 sdq8_5 ∼∼ sdq8_6

 sdq8_5 ∼∼ sdq8_7

 sdq8_5 ∼∼ sdq8_8

 sdq8_5 ∼∼ sdq8_9

 sdq8_6 ∼∼ sdq8_7

 sdq8_6 ∼∼ sdq8_8

 sdq8_6 ∼∼ sdq8_9

 sdq8_7 ∼∼ sdq8_8

 sdq8_7 ∼∼ sdq8_9

 sdq8_8 ∼∼ sdq8_9

# item 13 all time points

 sdq13_4 ∼∼ sdq13_5

 sdq13_4 ∼∼ sdq13_6

 sdq13_4 ∼∼ sdq13_7

 sdq13_4 ∼∼ sdq13_8

 sdq13_4 ∼∼ sdq13_9

 sdq13_5 ∼∼ sdq13_6

 sdq13_5 ∼∼ sdq13_7

 sdq13_5 ∼∼ sdq13_8

 sdq13_5 ∼∼ sdq13_9

 sdq13_6 ∼∼ sdq13_7

 sdq13_6 ∼∼ sdq13_8

 sdq13_6 ∼∼ sdq13_9

 sdq13_7 ∼∼ sdq13_8

 sdq13_7 ∼∼ sdq13_9

 sdq13_8 ∼∼ sdq13_9

# item 16 all time points

 sdq16_4 ∼∼ sdq16_5

 sdq16_4 ∼∼ sdq16_6

 sdq16_4 ∼∼ sdq16_7

 sdq16_4 ∼∼ sdq16_8

 sdq16_4 ∼∼ sdq16_9

 sdq16_5 ∼∼ sdq16_6

 sdq16_5 ∼∼ sdq16_7

 sdq16_5 ∼∼ sdq16_8

 sdq16_5 ∼∼ sdq16_9

 sdq16_6 ∼∼ sdq16_7

 sdq16_6 ∼∼ sdq16_8

 sdq16_6 ∼∼ sdq16_9

 sdq16_7 ∼∼ sdq16_8

 sdq16_7 ∼∼ sdq16_9

 sdq16_8 ∼∼ sdq16_9

# item 24 all time points

 sdq24_4 ∼∼ sdq24_5

 sdq24_4 ∼∼ sdq24_6

 sdq24_4 ∼∼ sdq24_7

 sdq24_4 ∼∼ sdq24_8

 sdq24_4 ∼∼ sdq24_9

 sdq24_5 ∼∼ sdq24_6

 sdq24_5 ∼∼ sdq24_7

 sdq24_5 ∼∼ sdq24_8

 sdq24_5 ∼∼ sdq24_9

 sdq24_6 ∼∼ sdq24_7

 sdq24_6 ∼∼ sdq24_8

 sdq24_6 ∼∼ sdq24_9

 sdq24_7 ∼∼ sdq24_8

 sdq24_7 ∼∼ sdq24_9

 sdq24_8 ∼∼ sdq24_9

"

# Fit the model

conf_emo_fit <- cfa(conf_emo, data = dataemo,

      estimator = "WLSMV", mimic = "mplus",

      parameterization = "theta",

      missing = "pairwise", ordered = dataemo)

# ––––––––––––––––––

# Scalar invariance emotional (m2b)

# ––––––––––––––––––

scalar_emo <- "

# Constraints are needed for longitudinal measurement invariance

# Factor models for emotional at 6 waves (constrained).

FY4 =∼ f*sdq3_4 + g*sdq8_4 + h*sdq13_4 + i*sdq16_4 + j*sdq24_4

FY5 =∼ f*sdq3_5 + g*sdq8_5 + h*sdq13_5 + i*sdq16_5 + j*sdq24_5

FY6 =∼ f*sdq3_6 + g*sdq8_6 + h*sdq13_6 + i*sdq16_6 + j*sdq24_6

FY7 =∼ f*sdq3_7 + g*sdq8_7 + h*sdq13_7 + i*sdq16_7 + j*sdq24_7

FY8 =∼ f*sdq3_8 + g*sdq8_8 + h*sdq13_8 + i*sdq16_8 + j*sdq24_8

FY9 =∼ f*sdq3_9 + g*sdq8_9 + h*sdq13_9 + i*sdq16_9 + j*sdq24_9

# threshold constraints emotional

sdq3_4 | f1*t1 + f2*t2

sdq3_5 | f1*t1 + f2*t2

sdq3_6 | f1*t1 + f2*t2

sdq3_7 | f1*t1 + f2*t2

sdq3_8 | f1*t1 + f2*t2

sdq3_9 | f1*t1 + f2*t2

sdq8_4 | g1*t1 + g2*t2

sdq8_5 | g1*t1 + g2*t2

sdq8_6 | g1*t1 + g2*t2

sdq8_7 | g1*t1 + g2*t2

sdq8_8 | g1*t1 + g2*t2

sdq8_9 | g1*t1 + g2*t2

sdq13_4 | h1*t1 + h2*t2

sdq13_5 | h1*t1 + h2*t2

sdq13_6 | h1*t1 + h2*t2

sdq13_7 | h1*t1 + h2*t2

sdq13_8 | h1*t1 + h2*t2

sdq13_9 | h1*t1 + h2*t2

sdq16_4 | i1*t1 + i2*t2

sdq16_5 | i1*t1 + i2*t2

sdq16_6 | i1*t1 + i2*t2

sdq16_7 | i1*t1 + i2*t2

sdq16_8 | i1*t1 + i2*t2

sdq16_9 | i1*t1 + i2*t2

sdq24_4 | j1*t1 + j2*t2

sdq24_5 | j1*t1 + j2*t2

sdq24_6 | j1*t1 + j2*t2

sdq24_7 | j1*t1 + j2*t2

sdq24_8 | j1*t1 + j2*t2

sdq24_9 | j1*t1 + j2*t2

# Covariances (observed emotional)

# item 3 all time points

 sdq3_4 ∼∼ sdq3_5

 sdq3_4 ∼∼ sdq3_6

 sdq3_4 ∼∼ sdq3_7

 sdq3_4 ∼∼ sdq3_8

 sdq3_4 ∼∼ sdq3_9

 sdq3_5 ∼∼ sdq3_6

 sdq3_5 ∼∼ sdq3_7

 sdq3_5 ∼∼ sdq3_8

 sdq3_5 ∼∼ sdq3_9

 sdq3_6 ∼∼ sdq3_7

 sdq3_6 ∼∼ sdq3_8

 sdq3_6 ∼∼ sdq3_9

 sdq3_7 ∼∼ sdq3_8

 sdq3_7 ∼∼ sdq3_9

 sdq3_8 ∼∼ sdq3_9

# item 8 all time points

 sdq8_4 ∼∼ sdq8_5

 sdq8_4 ∼∼ sdq8_6

 sdq8_4 ∼∼ sdq8_7

 sdq8_4 ∼∼ sdq8_8

 sdq8_4 ∼∼ sdq8_9

 sdq8_5 ∼∼ sdq8_6

 sdq8_5 ∼∼ sdq8_7

 sdq8_5 ∼∼ sdq8_8

 sdq8_5 ∼∼ sdq8_9

 sdq8_6 ∼∼ sdq8_7

 sdq8_6 ∼∼ sdq8_8

 sdq8_6 ∼∼ sdq8_9

 sdq8_7 ∼∼ sdq8_8

 sdq8_7 ∼∼ sdq8_9

 sdq8_8 ∼∼ sdq8_9

# item 13 all time points

 sdq13_4 ∼∼ sdq13_5

 sdq13_4 ∼∼ sdq13_6

 sdq13_4 ∼∼ sdq13_7

 sdq13_4 ∼∼ sdq13_8

 sdq13_4 ∼∼ sdq13_9

 sdq13_5 ∼∼ sdq13_6

 sdq13_5 ∼∼ sdq13_7

 sdq13_5 ∼∼ sdq13_8

 sdq13_5 ∼∼ sdq13_9

 sdq13_6 ∼∼ sdq13_7

 sdq13_6 ∼∼ sdq13_8

 sdq13_6 ∼∼ sdq13_9

 sdq13_7 ∼∼ sdq13_8

 sdq13_7 ∼∼ sdq13_9

 sdq13_8 ∼∼ sdq13_9

# item 16 all time points

 sdq16_4 ∼∼ sdq16_5

 sdq16_4 ∼∼ sdq16_6

 sdq16_4 ∼∼ sdq16_7

 sdq16_4 ∼∼ sdq16_8

 sdq16_4 ∼∼ sdq16_9

 sdq16_5 ∼∼ sdq16_6

 sdq16_5 ∼∼ sdq16_7

 sdq16_5 ∼∼ sdq16_8

 sdq16_5 ∼∼ sdq16_9

 sdq16_6 ∼∼ sdq16_7

 sdq16_6 ∼∼ sdq16_8

 sdq16_6 ∼∼ sdq16_9

 sdq16_7 ∼∼ sdq16_8

 sdq16_7 ∼∼ sdq16_9

 sdq16_8 ∼∼ sdq16_9

# item 24 all time points

 sdq24_4 ∼∼ sdq24_5

 sdq24_4 ∼∼ sdq24_6

 sdq24_4 ∼∼ sdq24_7

 sdq24_4 ∼∼ sdq24_8

 sdq24_4 ∼∼ sdq24_9

 sdq24_5 ∼∼ sdq24_6

 sdq24_5 ∼∼ sdq24_7

 sdq24_5 ∼∼ sdq24_8

 sdq24_5 ∼∼ sdq24_9

 sdq24_6 ∼∼ sdq24_7

 sdq24_6 ∼∼ sdq24_8

 sdq24_6 ∼∼ sdq24_9

 sdq24_7 ∼∼ sdq24_8

 sdq24_7 ∼∼ sdq24_9

 sdq24_8 ∼∼ sdq24_9

"

#Fit the model

scalar_emo_fit <- cfa(scalar_emo, data = dataemo,

      estimator = "WLSMV", mimic = "mplus",

      parameterization = "theta",

      missing = "pairwise", ordered = dataemo)

## Appendix 3 Syntax for growth mixture models

These models were fitted using the following code in Latent Gold 6.0. Comments are preceded by “//”. Indentation is not required.

***Longitudinal poverty:***

***Longitudinal work intensity:***

## Appendix 4 Syntax for Bivariate Growth curve model

The model in equation A.1 can be run using the R package “R2MLwiN” [15] with the following syntax. Comments are preceded by “#” and indentation is not required.

Equation A.1 is an unconditional non-linear growth curve. To fit a conditional growth curve model, further variables need to be incorporated into the fixed part of the model, separated by “+” sign. Interaction effects are easily added by placing a “*” sign between the main effects of interest. For example, the code below is to fit a model akin to the full model in Treanor and Troncoso [11].
